# Supplementary figures and images for: DNA isolation protocol effects on nuclear DNA analysis by microarrays, droplet digital PCR, and whole genome sequencing, and on mitochondrial DNA copy number estimation
Source: PLoS One. 2017 Jul 6;12(7):e0180467. doi: 10.1371/journal.pone.0180467 (PMC5500342; doi:10.1371/journal.pone.0180467)

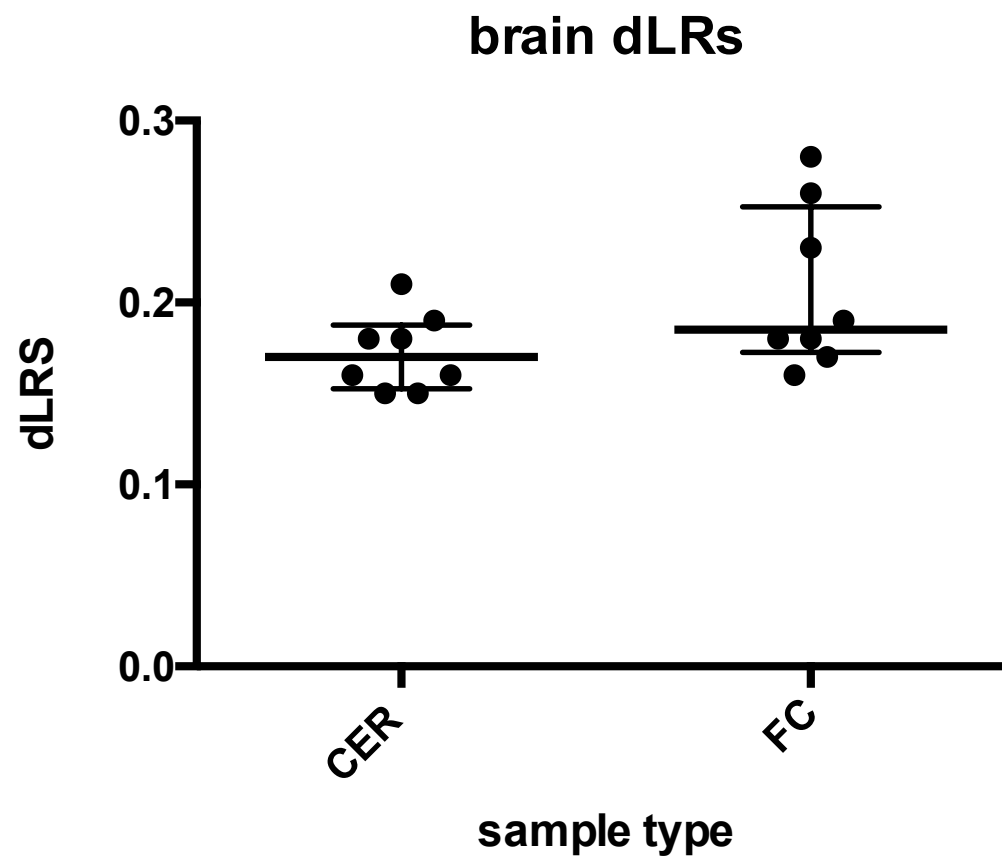

Supplement: S1 Fig — CER = cerebellum, FC = frontal cortex. The median and interquartile ranges are shown. (PDF) [file pone.0180467.s001.pdf]

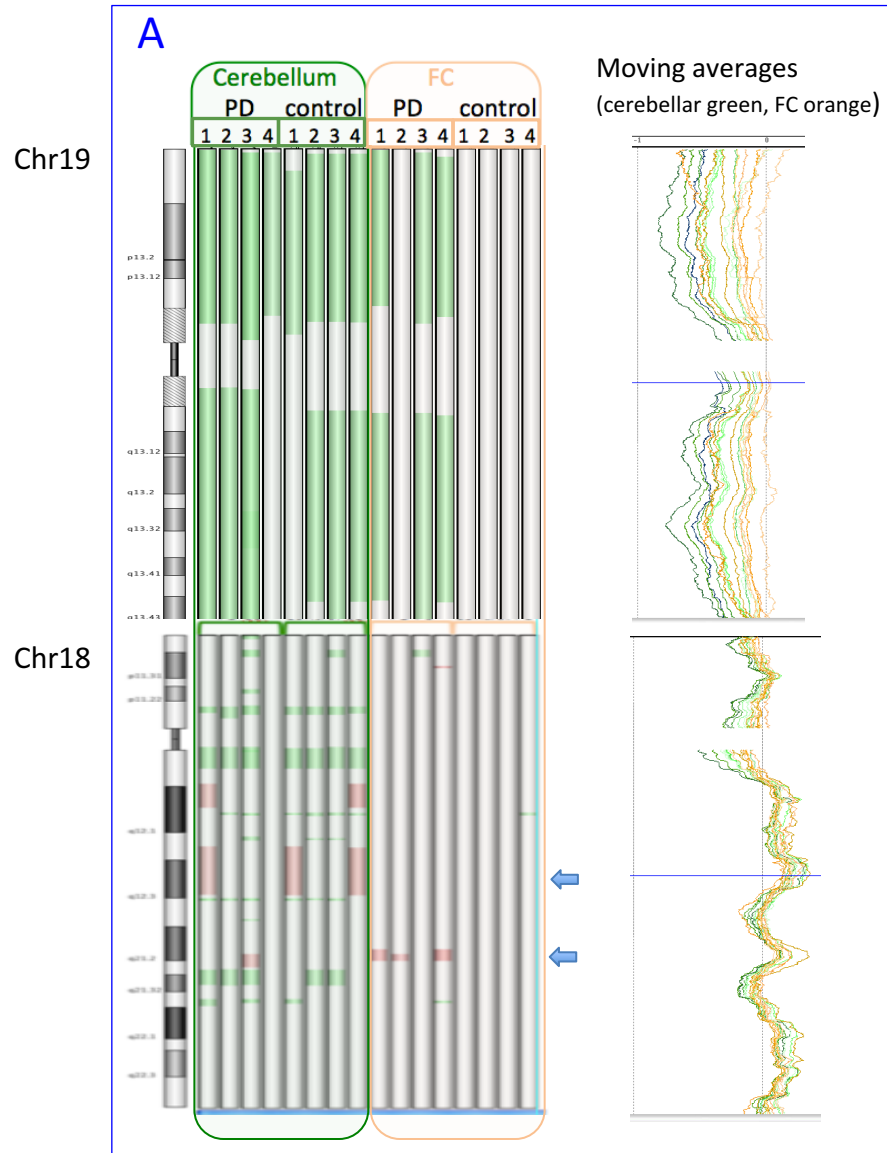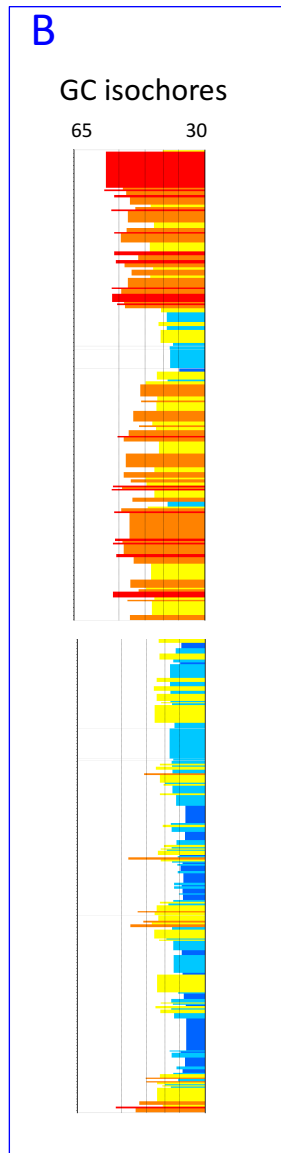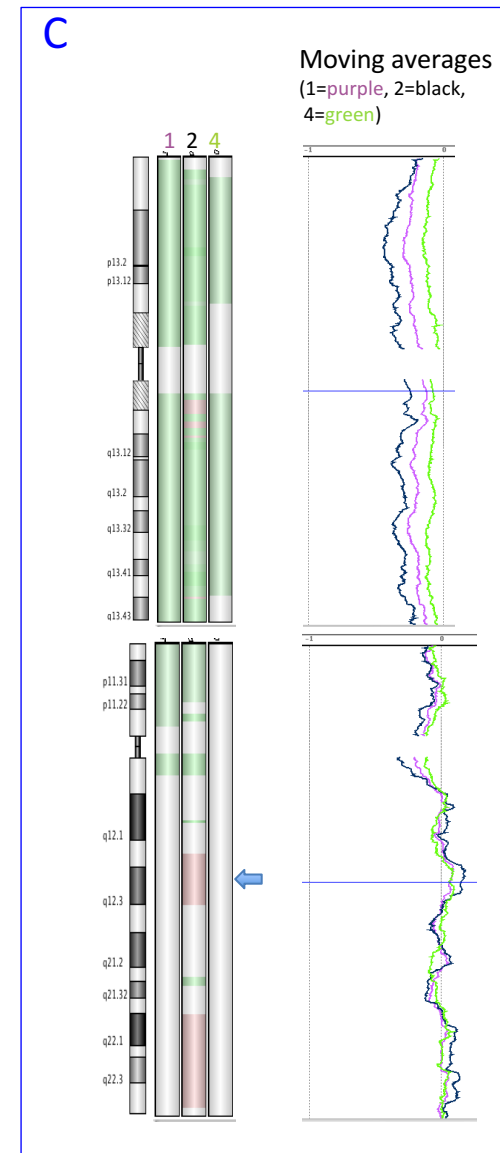

Supplement: S3 Fig — Analysis by ADM2 (FZ off). The ADM2 threshold is 12 for chr.19, and 6 for chr.18, as most changes were not visible at higher thresholds. 5 Mb moving averages are shown (a) Cerebellum and FC DNA with PBL DNA as reference. Arrows show gains in low GC regions.(b) The human GC content isochore plot (orange = high, blue = low; range 30–65%).(c) PD cerebellar DNA with FC from same brain as reference for PD1, 2, and 4. For PD2, analysis of the combined dye-flip pair is shown. Note that for chr18, in the arrowed low GC region, even the two samples where gains were not called have a slightly positive moving average. (PDF) [file pone.0180467.s003.pdf]

Chr.1

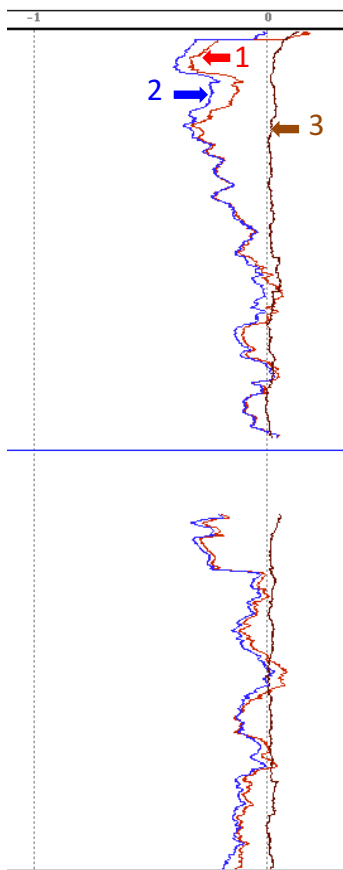

Chr.18

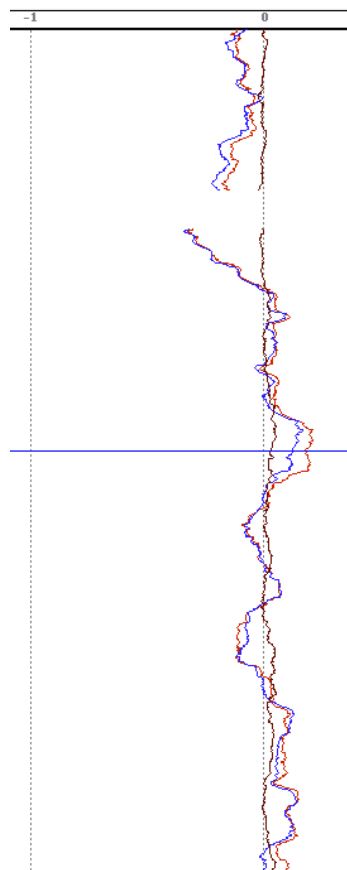

Chr.19

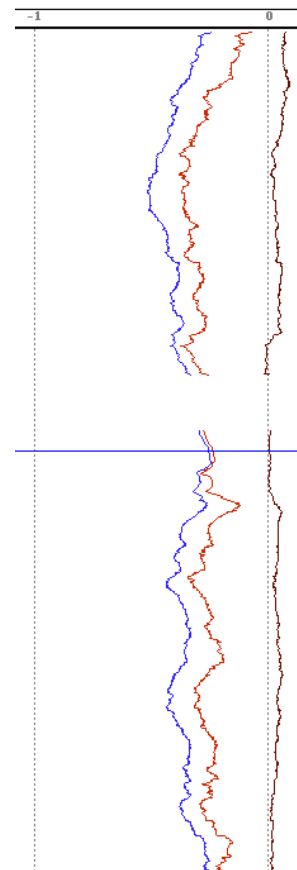

Supplement: S4 Fig — (1) Cerebellum (test) v FC (reference), red. (2) FC (test) v cerebellum (reference), dye flip specified during data import, blue. (3) Male to female reference PBL DNA hybridisation, brown, for comparison. Moving averages are shown over 10 Mb for chr.1, and 5 Mb for chr.18 and 19. (PDF) [file pone.0180467.s004.pdf]

Chr19

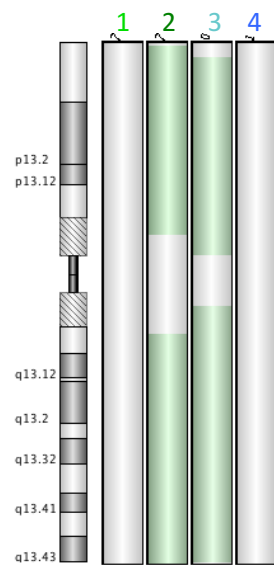

Moving averages

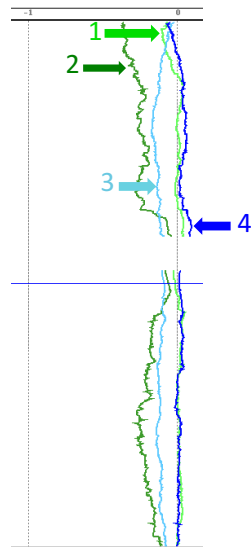

GC isochores

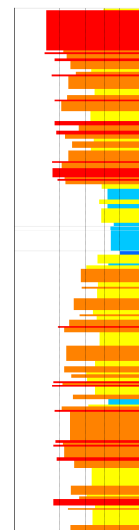

Chr18

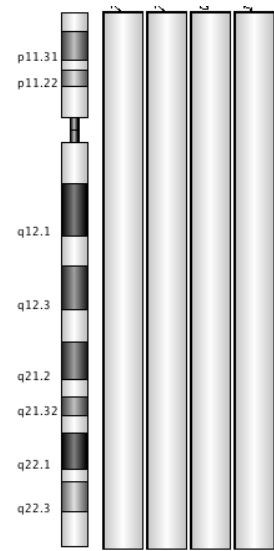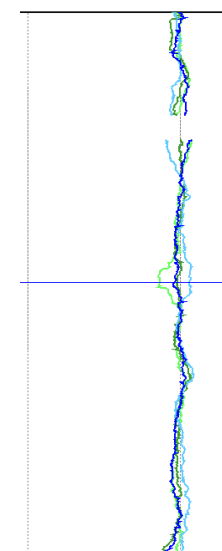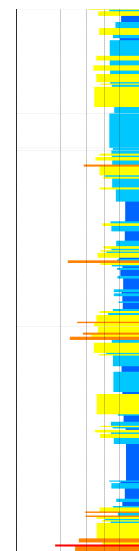

Supplement: S5 Fig — Analysis by ADM2 (FZ off, threshold 12 for chr.19, 6 for chr.18), with 5 Mb moving averages, and GC isochores; range 30–65%). (1–3): Hybridisations of spin column-extracted cerebellar DNA, with Puregene extracted DNA from same cerebellum as reference. (1) PD3, 5 mg spin column extraction; (2) PD3, 25mg spin column extraction; (3) PD4, 25 mg spin column extraction. (4) PD1, Puregene DNA, cerebellar, with FC as reference. Note the absence of waves and losses, unlike the same combination but after SC isolations, shown in S3C Fig, sample 1). (PDF) [file pone.0180467.s005.pdf]

A

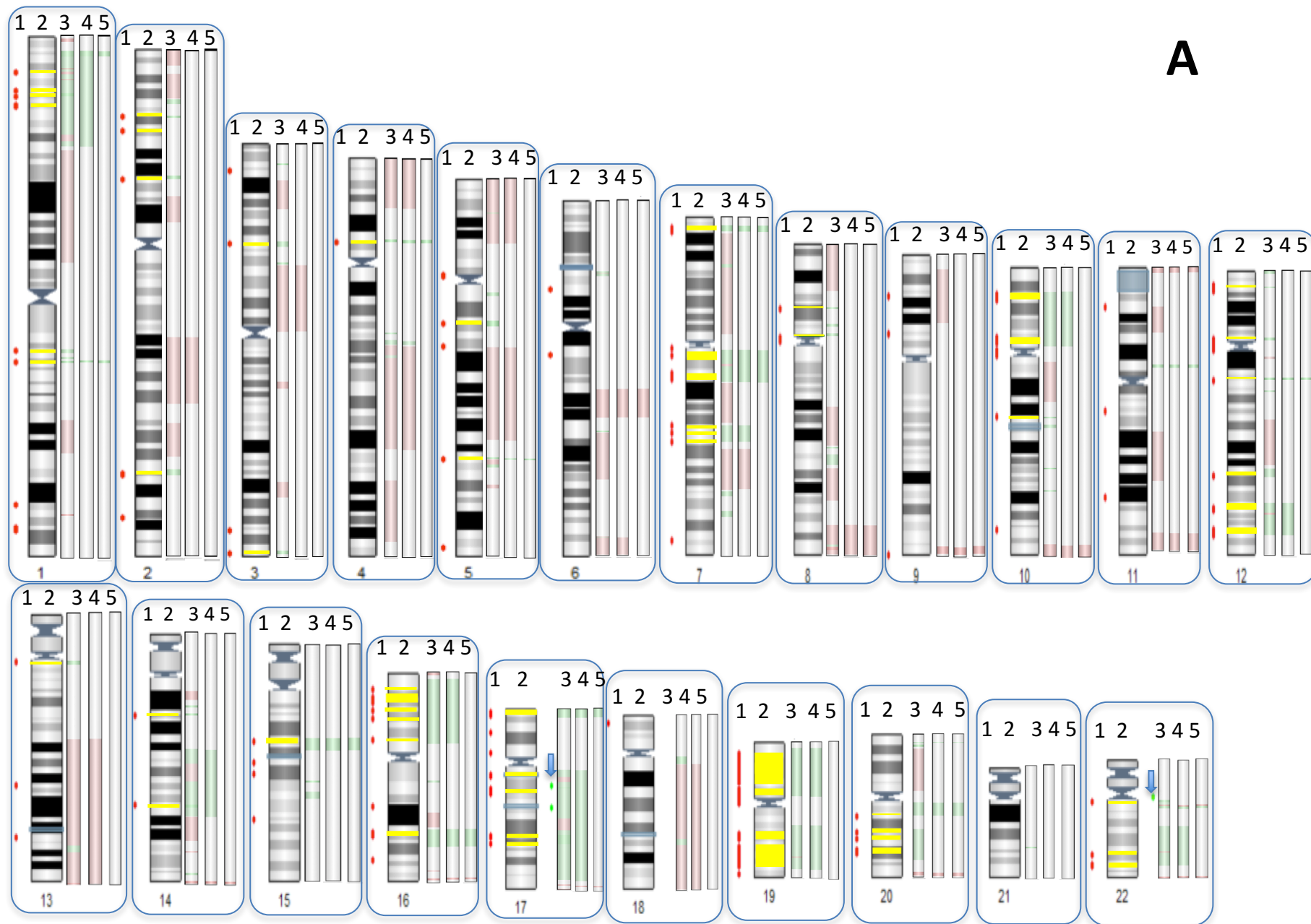

**B**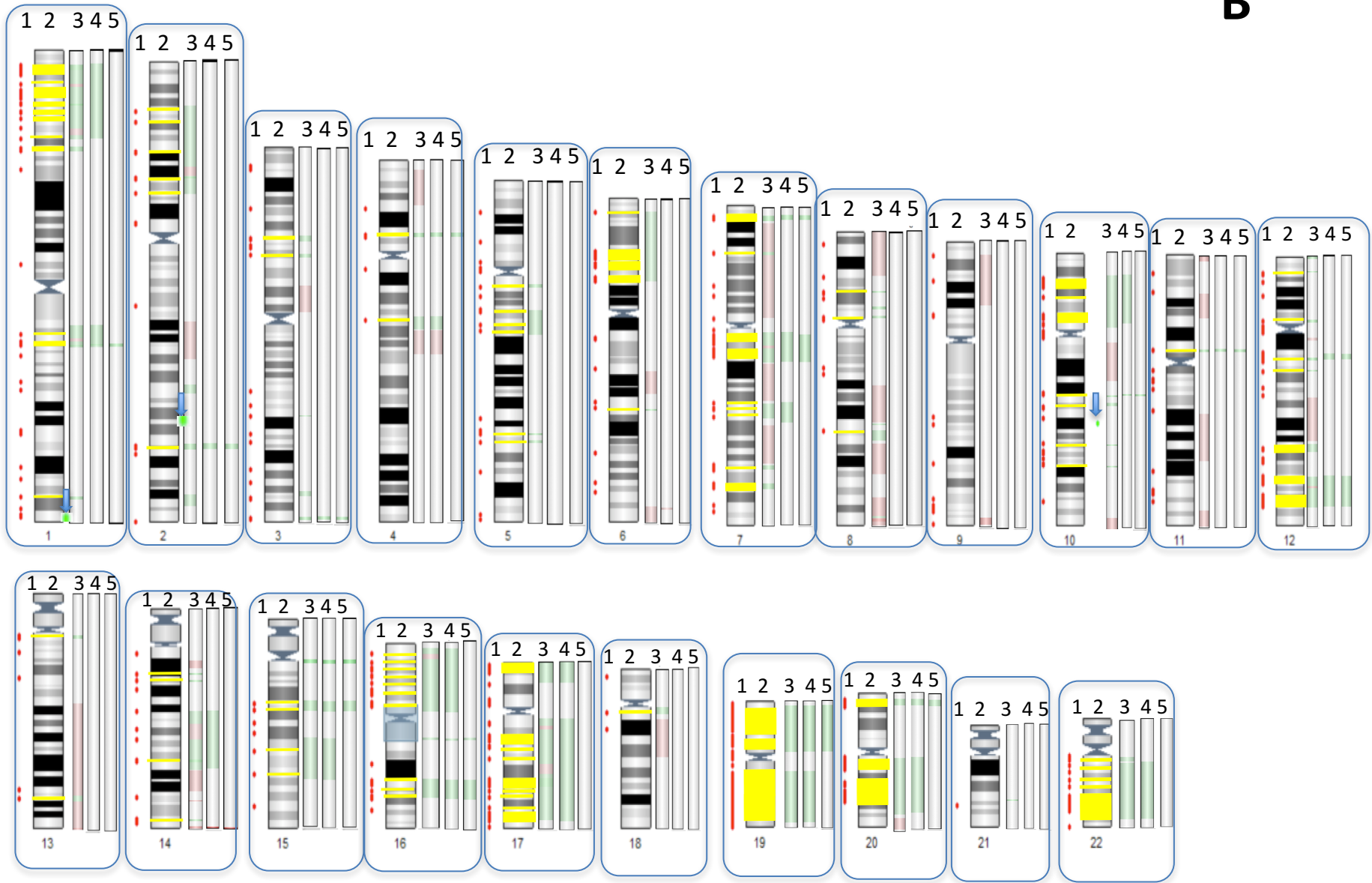

Supplement: S6 Fig — These samples had the highest number of losses on SNP array. The five columns for each chromosome are as follows: (1) Deletion calls by SNP array (red dots / bars)(2) Common deletion calls. Yellow lines / bars represent areas called as losses by SNP array and aCGH (using ADM2, threshold 8, FZ off)(3) aCGH (ADM2, threshold 8, FZ off). Losses are green, gains are red.(4) aCGH (ADM2, threshold 12, FZ off).(5) aCGH (ADM2, threshold 12, FZ off). An additional filter was used to filter calls that have a level of <15% gain or loss. Note that almost all the losses at these settings are also called by the SNP array. The rare gains on SNP array are shown as green dots between columns 2 and 3, and highlighted with a blue arrow. (PDF) [file pone.0180467.s006.pdf]

A

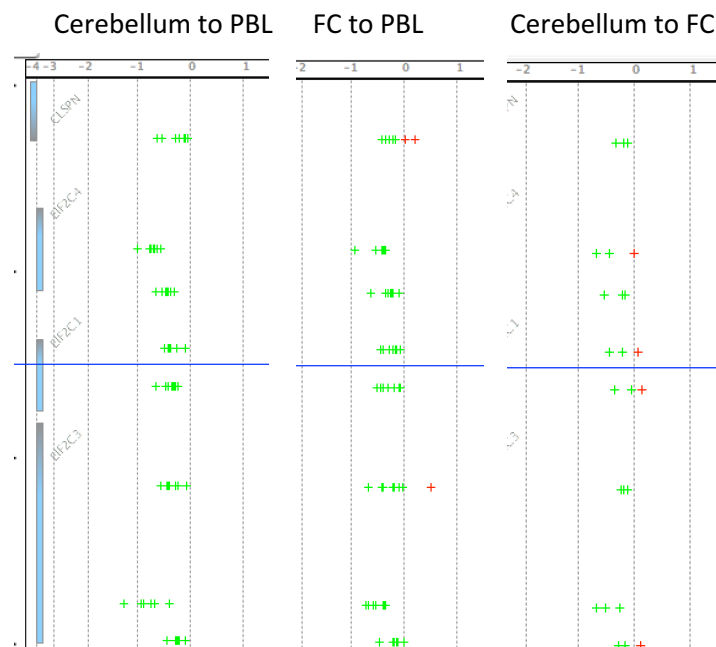

B

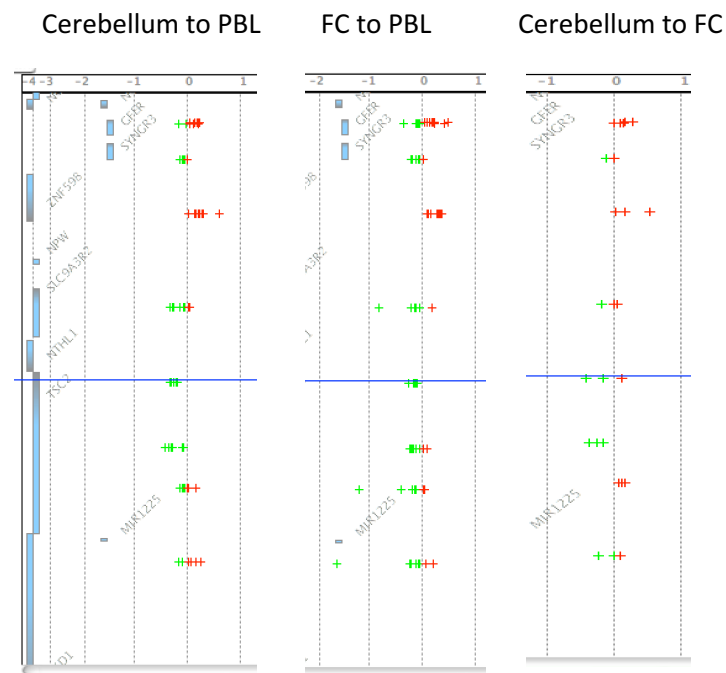

Supplement: S8 Fig — Probe dLRs in each hybridisation are shown, grouped by type, with ddPCR target location indicated by a blue line. For PD2 Cerebellum to FC, the combined the dye-flip data were used. (A) EIF2C1, 325 kb shown (chr1:36199314–3652540)(B) TSC2, 144 kb shown (chr16:2027153–2172009) (PDF) [file pone.0180467.s008.pdf]

A

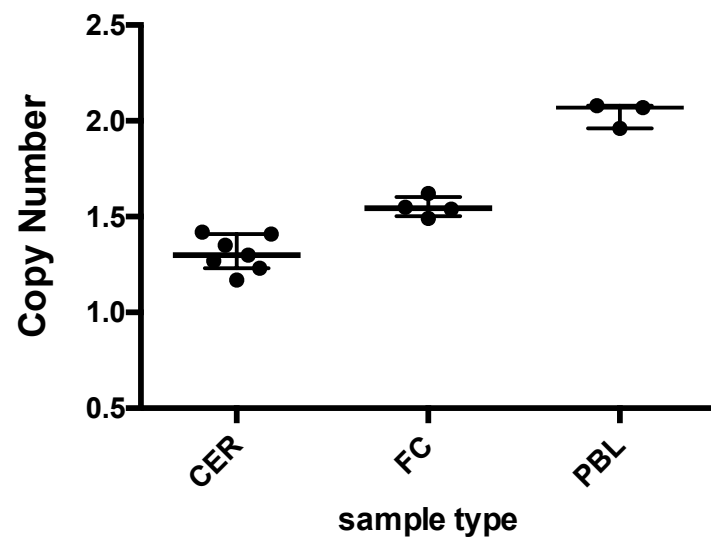

B

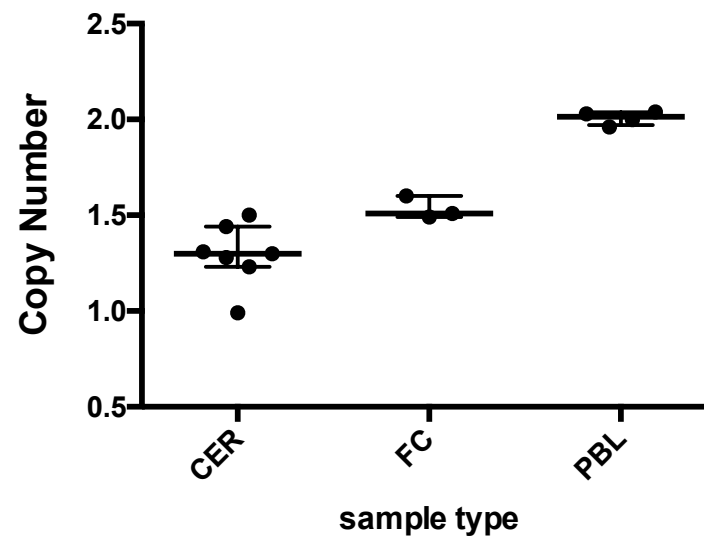

Supplement: S9 Fig — The median and interquartile ranges are shown in all cases. (a) EIF2C1. CER and FC from four brains, CER only from another three, and three control PBL DNA samples. Kruskal-Wallis p < 0.001 (b) TSC2. CER and FC from three brains, and cerebellum only from another four, and four control PBL DNA samples. Kruskal-Wallis p = 0.0001. (PDF) [file pone.0180467.s009.pdf]

Phenol / Chloroform

Puregene

PD5

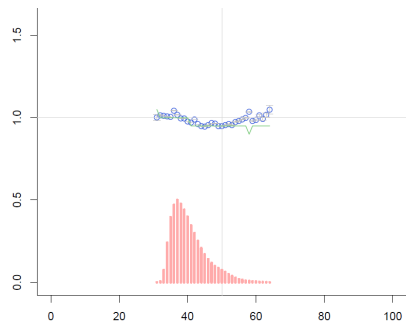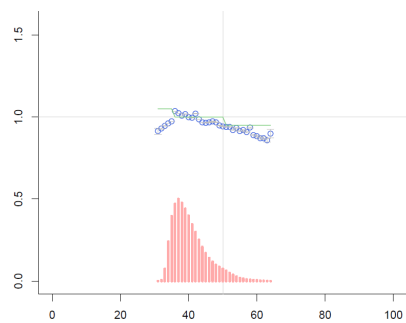

PD6

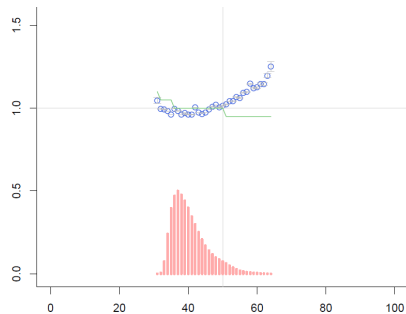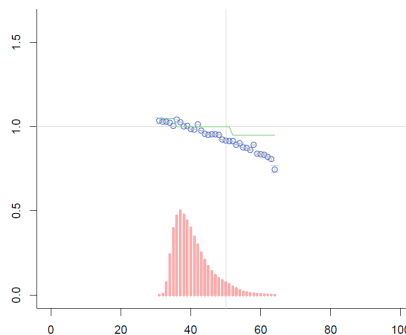

ILBD

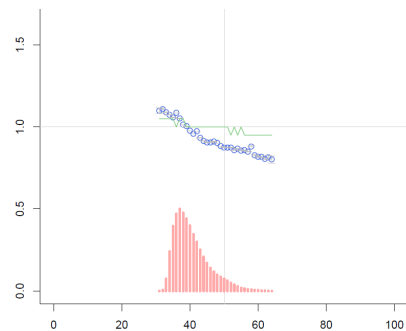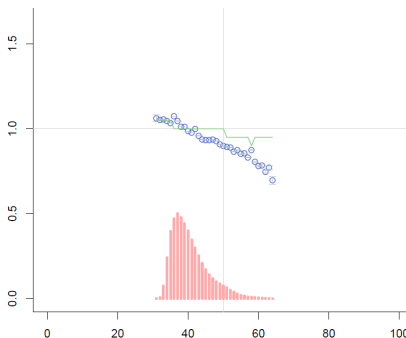

○ Normalized Coverage  
■ Windows at GC%  
— Base Quality at GC%

Supplement: S10 Fig — The mean normalized coverage per 100 kb window is shown (y-axis) and the % content of each window (x-axis). The base quality for each GC content is also shown. (PDF) [file pone.0180467.s010.pdf]

A

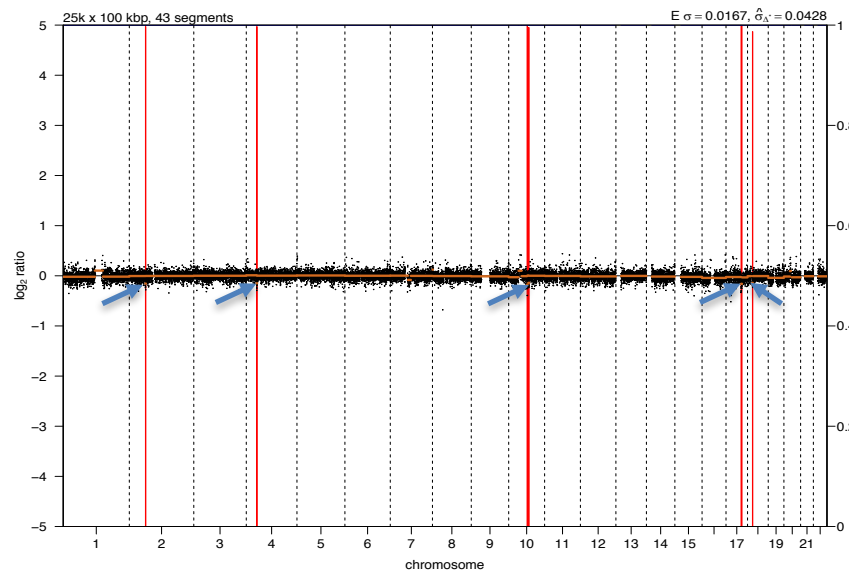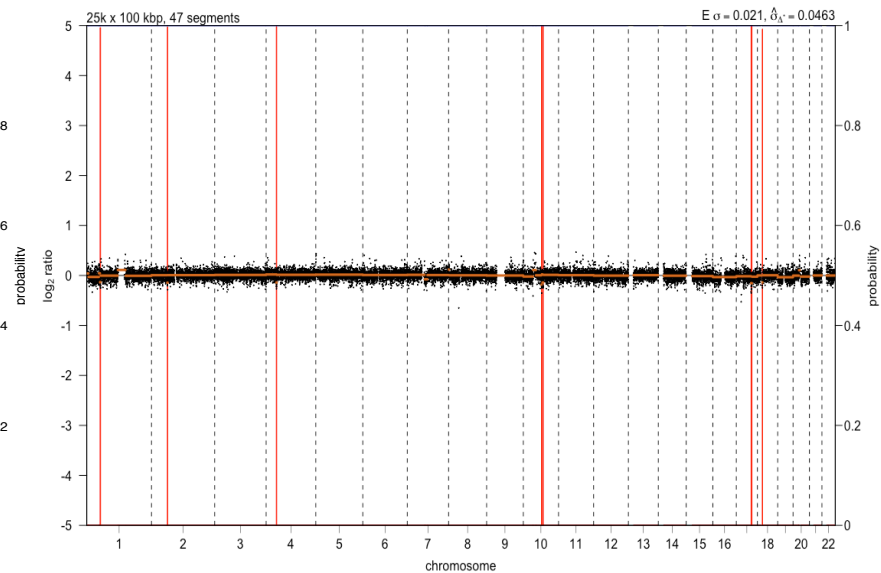

B

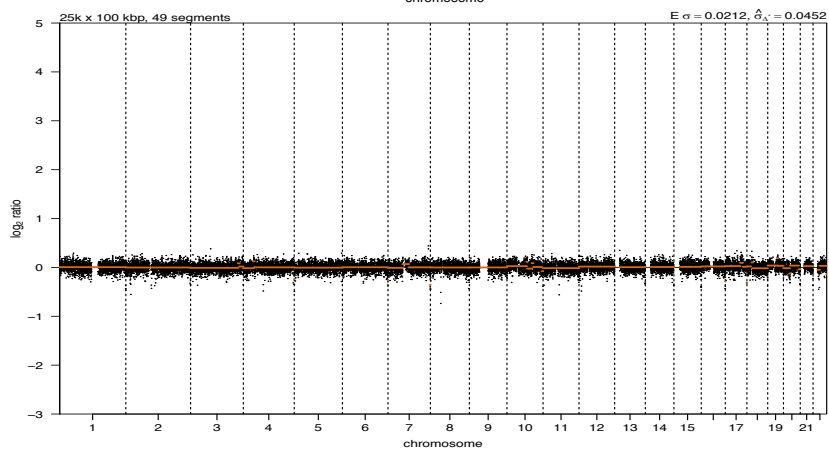

Supplement: S11 Fig — (A) Phenol / Chloroform. (B) Puregene. The total PE read number was 97,978,308 for SC, and 62,120,676 for Puregene. The right-hand figure in A shows the results after downsampling to 62,115,269 reads, done with Picard DownsampleSam (strategy = high accuracy). The estimated minimum standard deviation due purely to read counting (Eσ) and the observed standard deviation (σΔ) are shown. The y-axes show the log ratio (left) and probability assigned to the aberration called (right). The observed losses in A had a minimally negative log ratio, and are indicated by arrows for clarity. (PDF) [file pone.0180467.s011.pdf]

Chr1

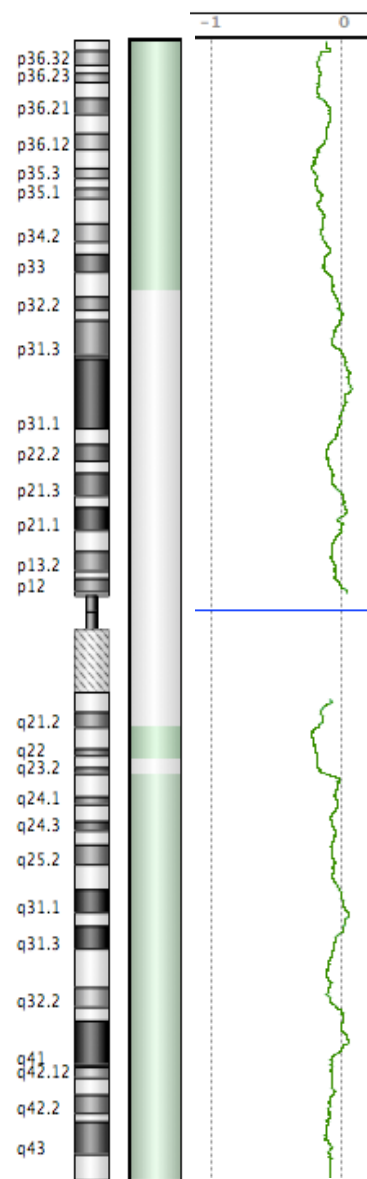

Chr19

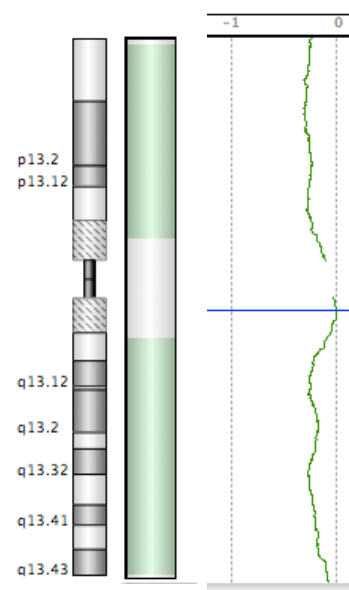

Chr18

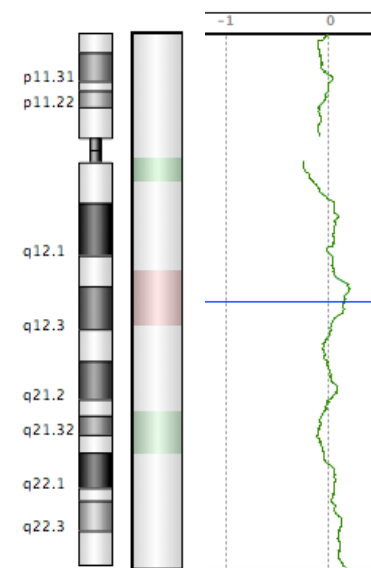

Supplement: S12 Fig — The moving averages are shown for chr1 (over 10 Mb), and 18 and 19 (5 Mb). Aberrations called with FZ off are highlighted (threshold 12 for chr1 and 19, 6 for chr18). (PDF) [file pone.0180467.s012.pdf]
