# Supplementary material for: DNA isolation protocol effects on nuclear DNA analysis by microarrays, droplet digital PCR, and whole genome sequencing, and on mitochondrial DNA copy number estimation
Source: PLoS One. 2017 Jul 6;12(7):e0180467. doi: 10.1371/journal.pone.0180467 (PMC5500342; doi:10.1371/journal.pone.0180467)
Supplement: S7 Fig — The left hand column shows the relevant data including with B allele frequency comparison to aCGH from C1 cerebellum. The right hand column shows the SNP logR over this region in selected other three samples where it was also somewhat negative, although losses were not always called. A. Loss around FBXO42 (chr1:16,619,350–16,773,880; 154.5 kb). This was examined further by PCR, and not confirmed (see S1 Note).B. Loss in 1q22 region (chr1: 155,540,660–155,819,657; 279 kb). (PDF) [file pone.0180467.s007.pdf]

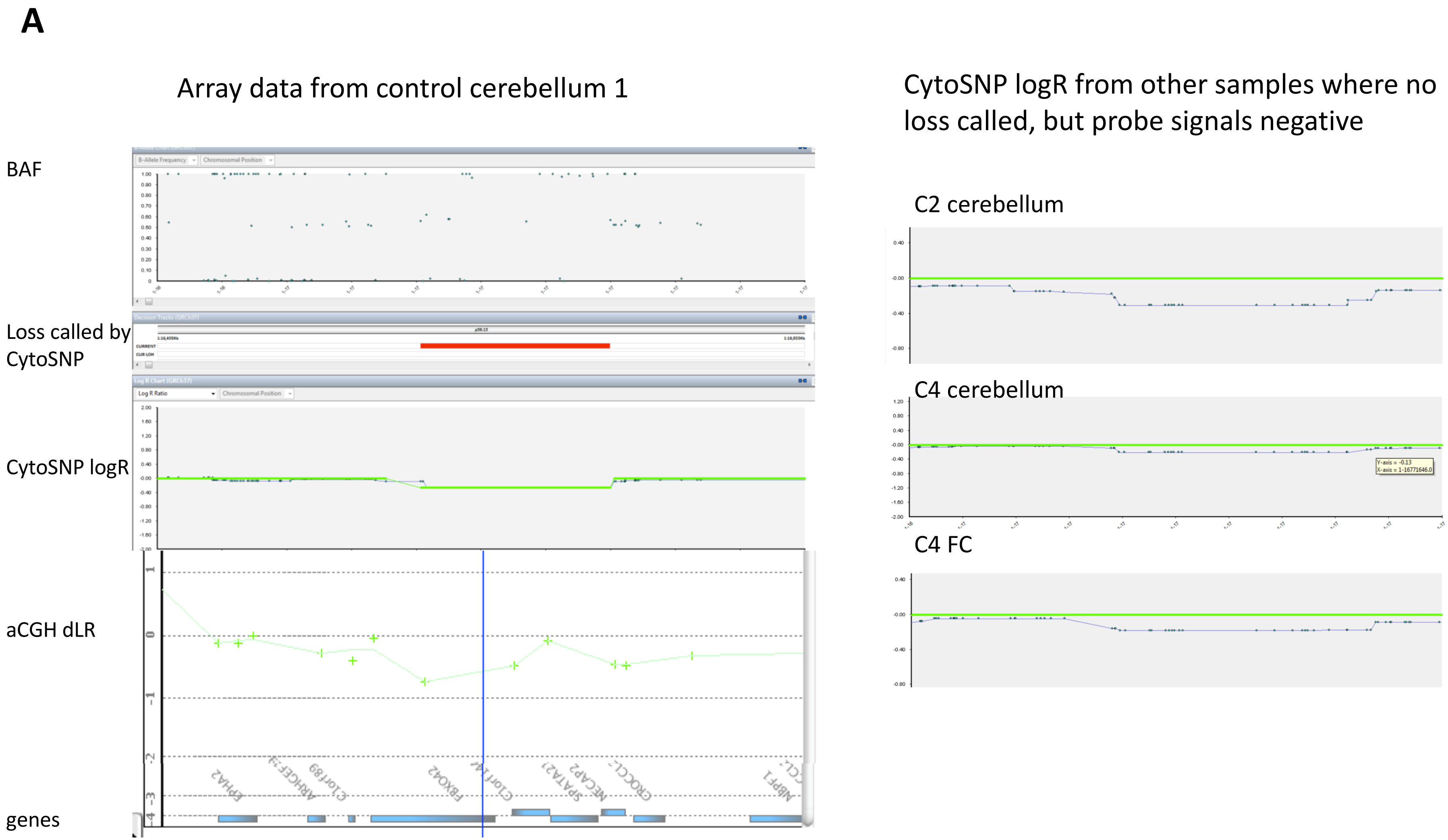
A
Array data from control cerebellum 1
CytoSNP logR from other samples where no loss called, but probe signals negative
BAF
Loss called by CytoSNP
CytoSNP logR
aCGH dLR
genes
C2 cerebellum
C4 cerebellum
C4 FC

B

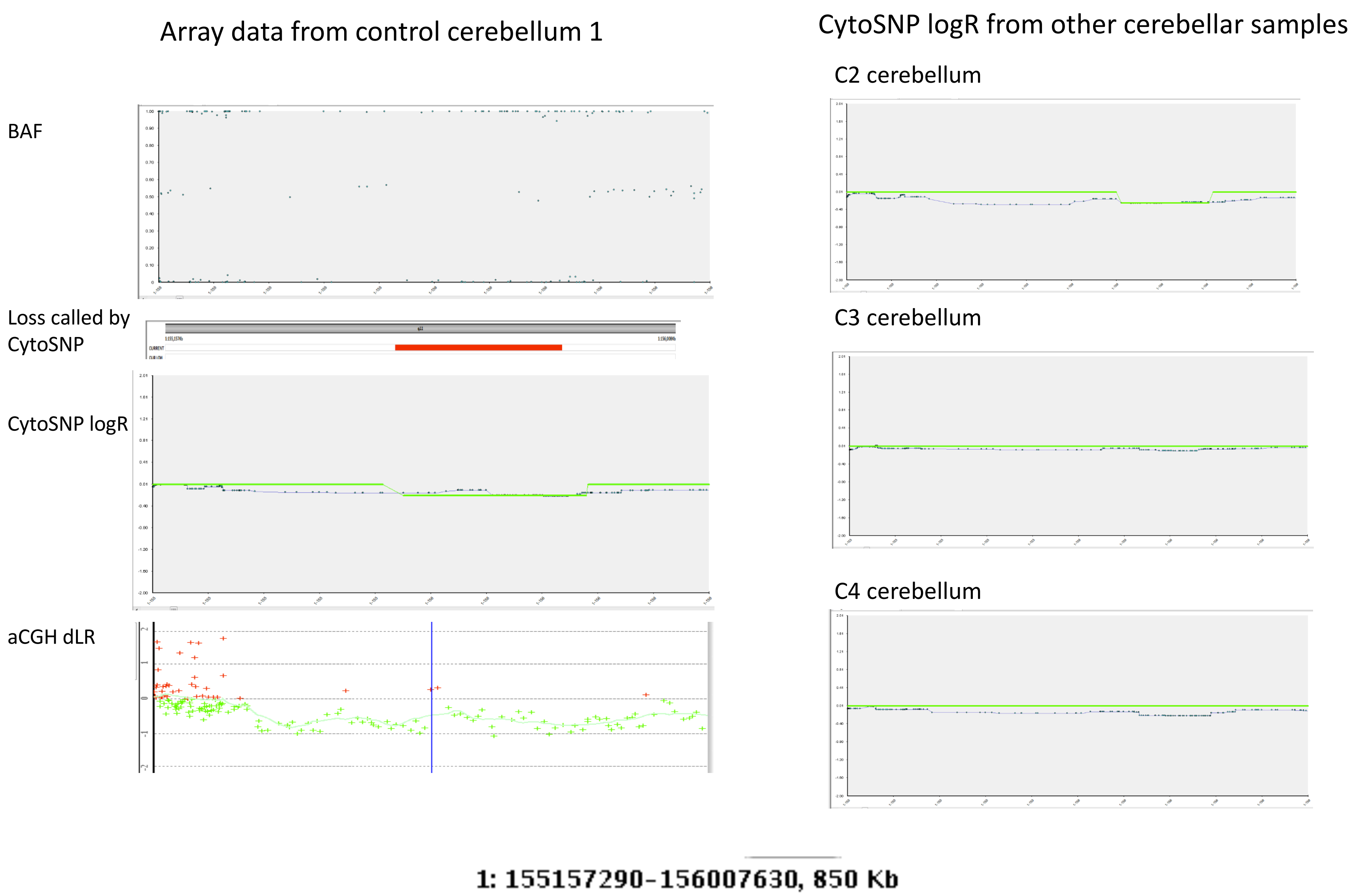
Array data from control cerebellum 1
CytoSNP logR from other cerebellar samples
BAF
Loss called by CytoSNP
CytoSNP logR
aCGH dLR
C2 cerebellum
C3 cerebellum
C4 cerebellum
1: 155157290-156007630, 850 Kb
