# Supplementary material for: DNA isolation protocol effects on nuclear DNA analysis by microarrays, droplet digital PCR, and whole genome sequencing, and on mitochondrial DNA copy number estimation
Source: PLoS One. 2017 Jul 6;12(7):e0180467. doi: 10.1371/journal.pone.0180467 (PMC5500342; doi:10.1371/journal.pone.0180467)
Supplement: S1 Table — CER = cerebellum. ON = overnight. S/C = spin column. PG = puregene. Ref = used as reference DNA in aCGH. Individual experiments explained in text. (PPTX) [file pone.0180467.s013.pptx]

## Slide 1
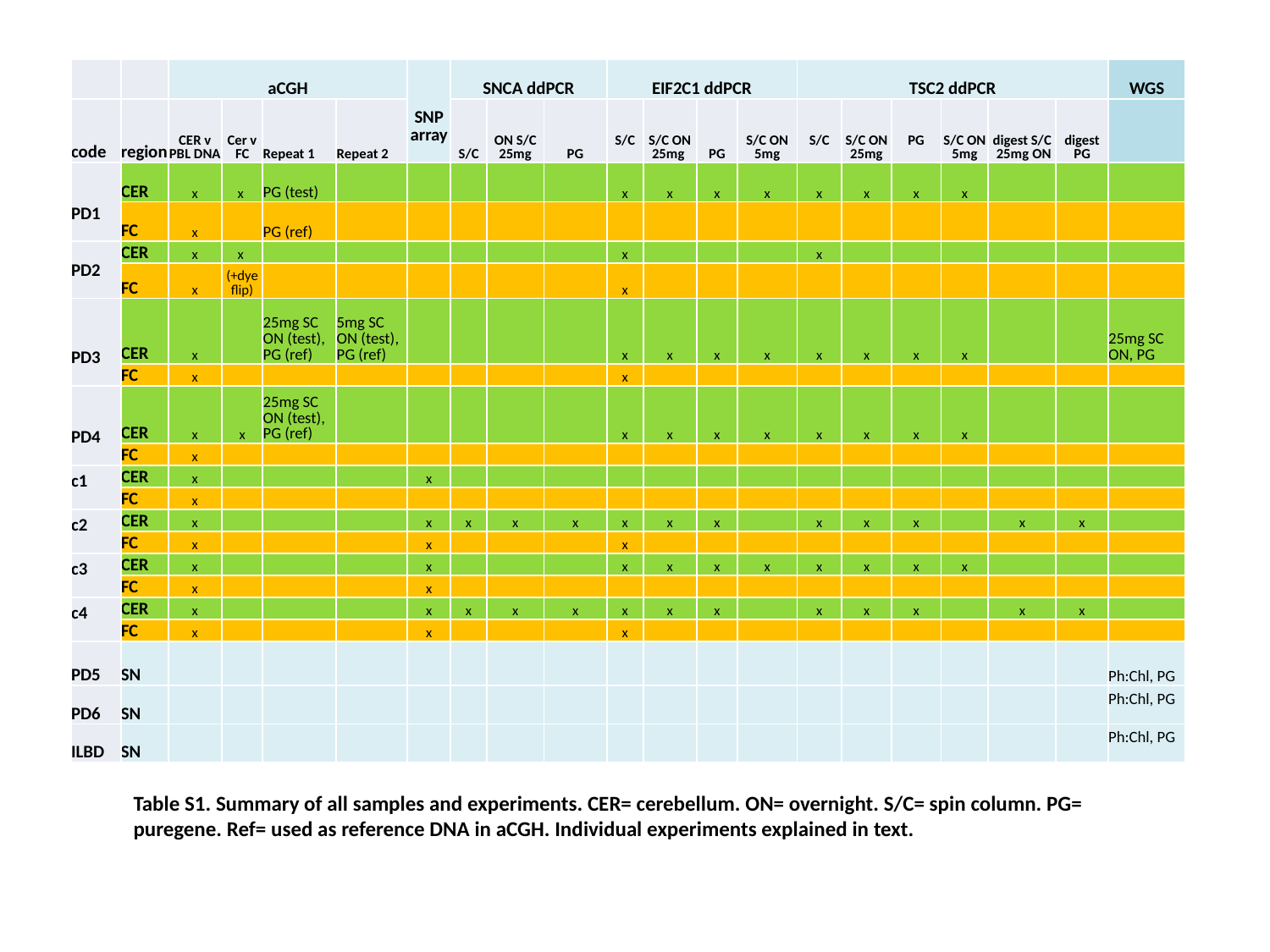

| | | aCGH | | | | SNP array | SNCA ddPCR | | | EIF2C1 ddPCR | | | | TSC2 ddPCR | | | | | | WGS |
| --- | --- | --- | --- | --- | --- | --- | --- | --- | --- | --- | --- | --- | --- | --- | --- | --- | --- | --- | --- | --- |
| code | region | CER v PBL DNA | Cer v FC | Repeat 1 | Repeat 2 | | S/C | ON S/C 25mg | PG | S/C | S/C ON 25mg | PG | S/C ON 5mg | S/C | S/C ON 25mg | PG | S/C ON 5mg | digest S/C 25mg ON | digest PG | |
| PD1 | CER | x | x | PG (test) | | | | | | x | x | x | x | x | x | x | x | | | |
| | FC | x | | PG (ref) | | | | | | | | | | | | | | | | |
| PD2 | CER | x | x | | | | | | | x | | | | x | | | | | | |
| | FC | x | (+dye flip) | | | | | | | x | | | | | | | | | | |
| PD3 | CER | x | | 25mg SC ON (test), PG (ref) | 5mg SC ON (test), PG (ref) | | | | | x | x | x | x | x | x | x | x | | | 25mg SC ON, PG |
| | FC | x | | | | | | | | x | | | | | | | | | | |
| PD4 | CER | x | x | 25mg SC ON (test), PG (ref) | | | | | | x | x | x | x | x | x | x | x | | | |
| | FC | x | | | | | | | | | | | | | | | | | | |
| c1 | CER | x | | | | x | | | | | | | | | | | | | | |
| | FC | x | | | | | | | | | | | | | | | | | | |
| c2 | CER | x | | | | x | x | x | x | x | x | x | | x | x | x | | x | x | |
| | FC | x | | | | x | | | | x | | | | | | | | | | |
| c3 | CER | x | | | | x | | | | x | x | x | x | x | x | x | x | | | |
| | FC | x | | | | x | | | | | | | | | | | | | | |
| c4 | CER | x | | | | x | x | x | x | x | x | x | | x | x | x | | x | x | |
| | FC | x | | | | x | | | | x | | | | | | | | | | |
| PD5 | SN | | | | | | | | | | | | | | | | | | | Ph:Chl, PG |
| PD6 | SN | | | | | | | | | | | | | | | | | | | Ph:Chl, PG |
| ILBD | SN | | | | | | | | | | | | | | | | | | | Ph:Chl, PG |
Table S1.
Table S1. Summary of all samples and experiments. CER= cerebellum. ON= overnight. S/C= spin column. PG= puregene. Ref= used as reference DNA in aCGH. Individual experiments explained in text.
