# Supplementary material for: DNA isolation protocol effects on nuclear DNA analysis by microarrays, droplet digital PCR, and whole genome sequencing, and on mitochondrial DNA copy number estimation
Source: PLoS One. 2017 Jul 6;12(7):e0180467. doi: 10.1371/journal.pone.0180467 (PMC5500342; doi:10.1371/journal.pone.0180467)
Supplement: S2 Table — The fragile site within which SNCA is located and its flanking regions were included (chr4:87–97 Mb), as constitutional CNVs may involve most of this. (PPTX) [file pone.0180467.s014.pptx]

## Slide 1
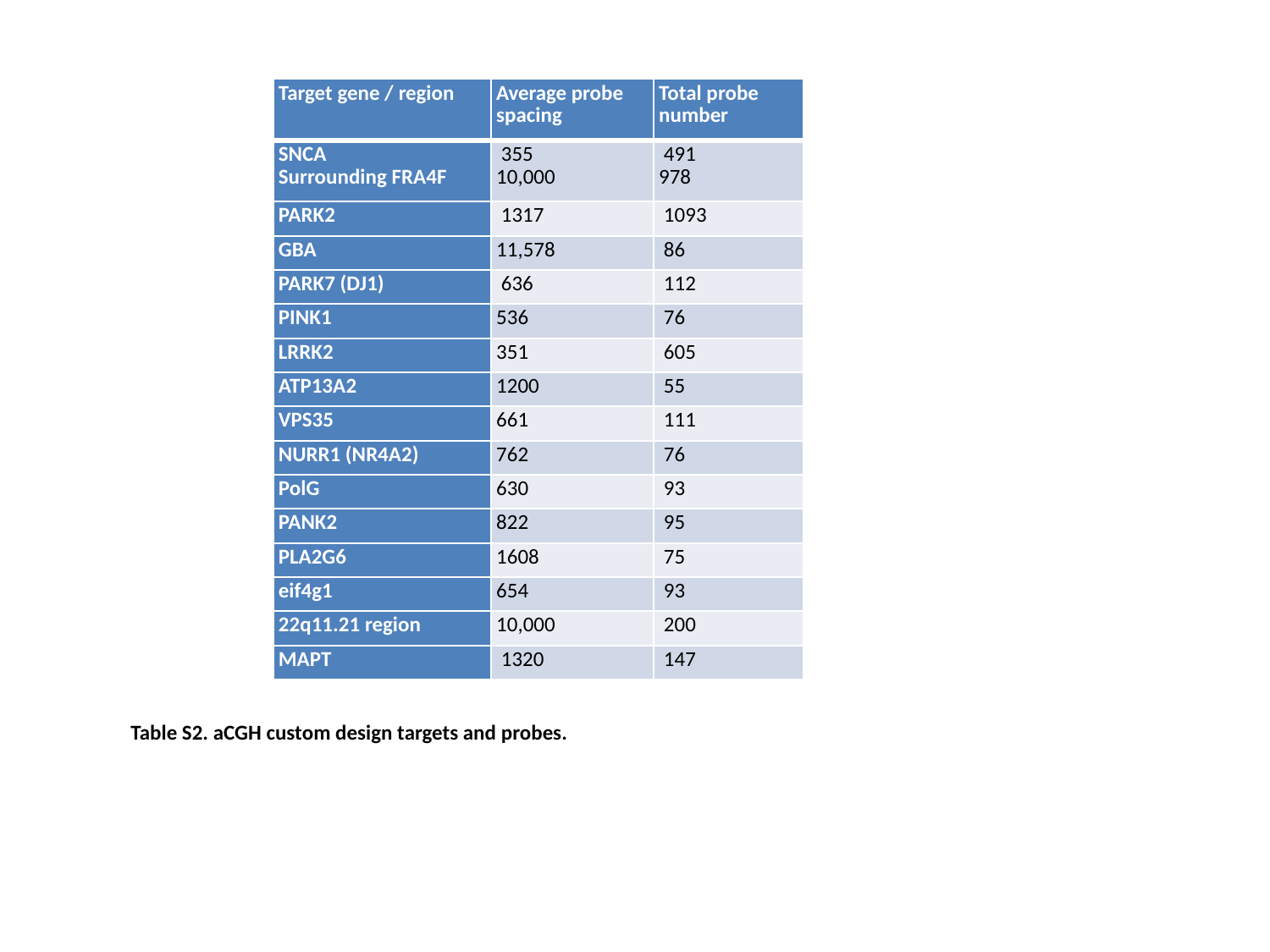

| Target gene / region | Average probe spacing | Total probe number |
| --- | --- | --- |
| SNCA Surrounding FRA4F | 355 10,000 | 491 978 |
| PARK2 | 1317 | 1093 |
| GBA | 11,578 | 86 |
| PARK7 (DJ1) | 636 | 112 |
| PINK1 | 536 | 76 |
| LRRK2 | 351 | 605 |
| ATP13A2 | 1200 | 55 |
| VPS35 | 661 | 111 |
| NURR1 (NR4A2) | 762 | 76 |
| PolG | 630 | 93 |
| PANK2 | 822 | 95 |
| PLA2G6 | 1608 | 75 |
| eif4g1 | 654 | 93 |
| 22q11.21 region | 10,000 | 200 |
| MAPT | 1320 | 147 |
Table S2. aCGH custom design targets and probes.
