# Supplementary material for: DNA isolation protocol effects on nuclear DNA analysis by microarrays, droplet digital PCR, and whole genome sequencing, and on mitochondrial DNA copy number estimation
Source: PLoS One. 2017 Jul 6;12(7):e0180467. doi: 10.1371/journal.pone.0180467 (PMC5500342; doi:10.1371/journal.pone.0180467)
Supplement: S3 Table — For each sample, the exact tissue mass used in spin column extractions (in mg), the DNA yield (in ng DNA per mg tissue), and the 260/280 ratio are shown. Yield mean and SD are shown at bottom. ANOVA for yield in four samples where all protocols were used: p = 0.039. (PPTX) [file pone.0180467.s015.pptx]

## Slide 1
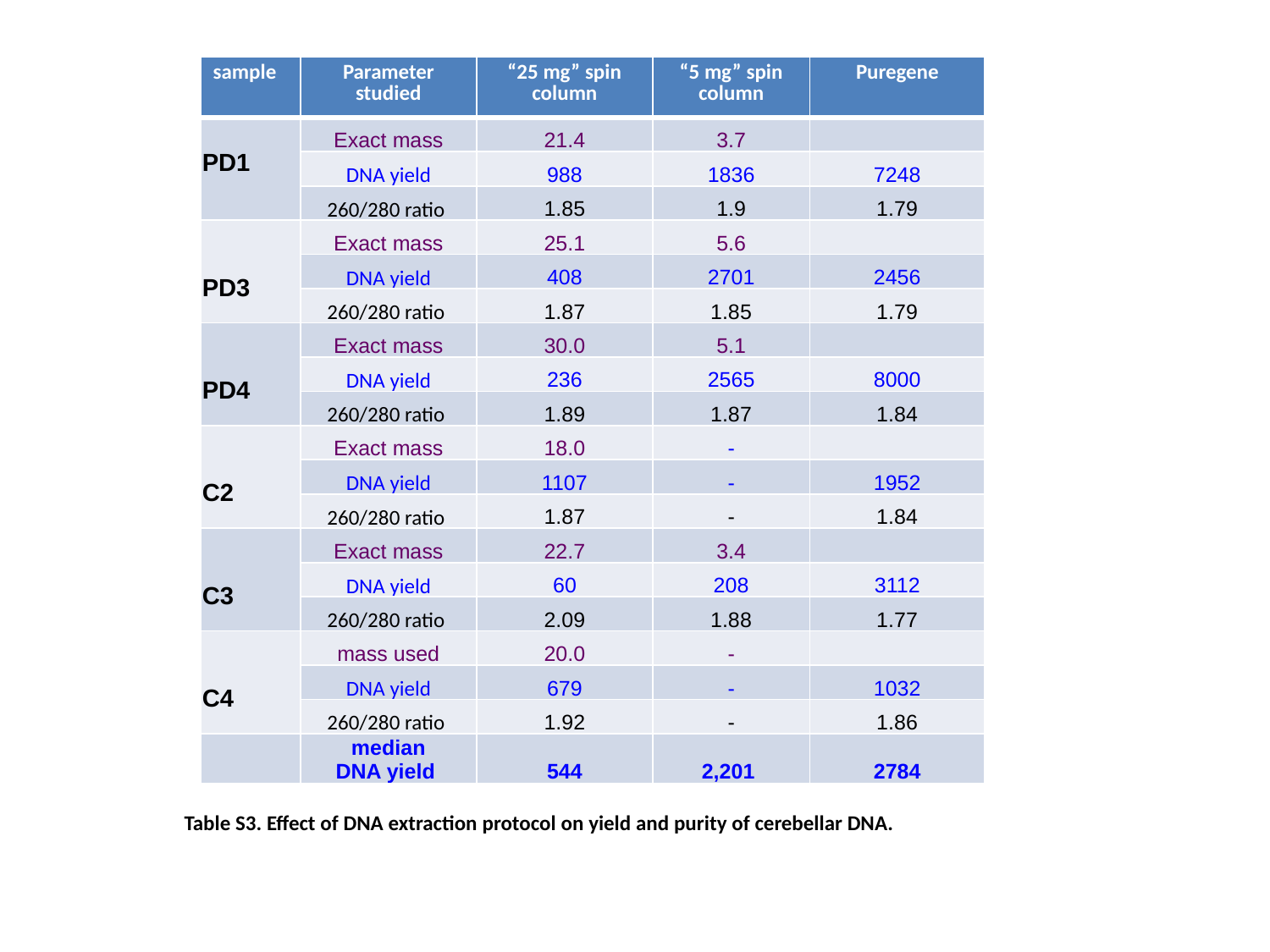

| sample | Parameter studied | “25 mg” spin column | “5 mg” spin column | Puregene |
| --- | --- | --- | --- | --- |
| PD1 | Exact mass | 21.4 | 3.7 | |
| | DNA yield | 988 | 1836 | 7248 |
| | 260/280 ratio | 1.85 | 1.9 | 1.79 |
| PD3 | Exact mass | 25.1 | 5.6 | |
| | DNA yield | 408 | 2701 | 2456 |
| | 260/280 ratio | 1.87 | 1.85 | 1.79 |
| PD4 | Exact mass | 30.0 | 5.1 | |
| | DNA yield | 236 | 2565 | 8000 |
| | 260/280 ratio | 1.89 | 1.87 | 1.84 |
| C2 | Exact mass | 18.0 | - | |
| | DNA yield | 1107 | - | 1952 |
| | 260/280 ratio | 1.87 | - | 1.84 |
| C3 | Exact mass | 22.7 | 3.4 | |
| | DNA yield | 60 | 208 | 3112 |
| | 260/280 ratio | 2.09 | 1.88 | 1.77 |
| C4 | mass used | 20.0 | - | |
| | DNA yield | 679 | - | 1032 |
| | 260/280 ratio | 1.92 | - | 1.86 |
| | median DNA yield | 544 | 2,201 | 2784 |
Table S3. Effect of DNA extraction protocol on yield and purity of cerebellar DNA.
