# Supplementary material for: DNA isolation protocol effects on nuclear DNA analysis by microarrays, droplet digital PCR, and whole genome sequencing, and on mitochondrial DNA copy number estimation
Source: PLoS One. 2017 Jul 6;12(7):e0180467. doi: 10.1371/journal.pone.0180467 (PMC5500342; doi:10.1371/journal.pone.0180467)
Supplement: S4 Table — (PPTX) [file pone.0180467.s016.pptx]

## Slide 1
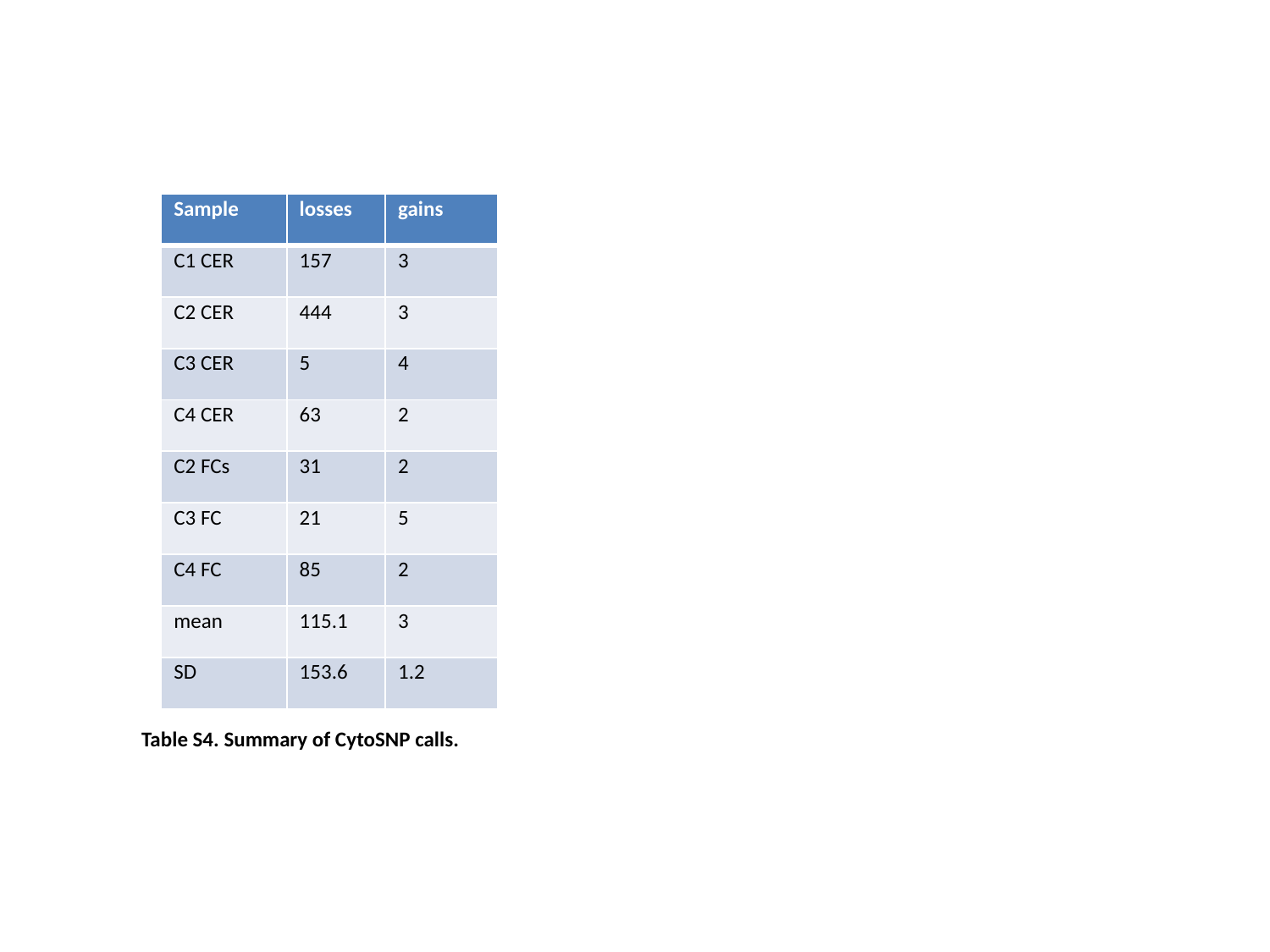

| Sample | losses | gains |
| --- | --- | --- |
| C1 CER | 157 | 3 |
| C2 CER | 444 | 3 |
| C3 CER | 5 | 4 |
| C4 CER | 63 | 2 |
| C2 FCs | 31 | 2 |
| C3 FC | 21 | 5 |
| C4 FC | 85 | 2 |
| mean | 115.1 | 3 |
| SD | 153.6 | 1.2 |
Table S4. Summary of CytoSNP calls.
