# Supplementary material for: DNA isolation protocol effects on nuclear DNA analysis by microarrays, droplet digital PCR, and whole genome sequencing, and on mitochondrial DNA copy number estimation
Source: PLoS One. 2017 Jul 6;12(7):e0180467. doi: 10.1371/journal.pone.0180467 (PMC5500342; doi:10.1371/journal.pone.0180467)
Supplement: S5 Table — Two control cerebellar samples analysed, with the standard protocol, and with restriction digestion as a separate step (“pre-digested”), in DNA extracted with spin columns overnight, or Puregene. (PPTX) [file pone.0180467.s017.pptx]

## Slide 1
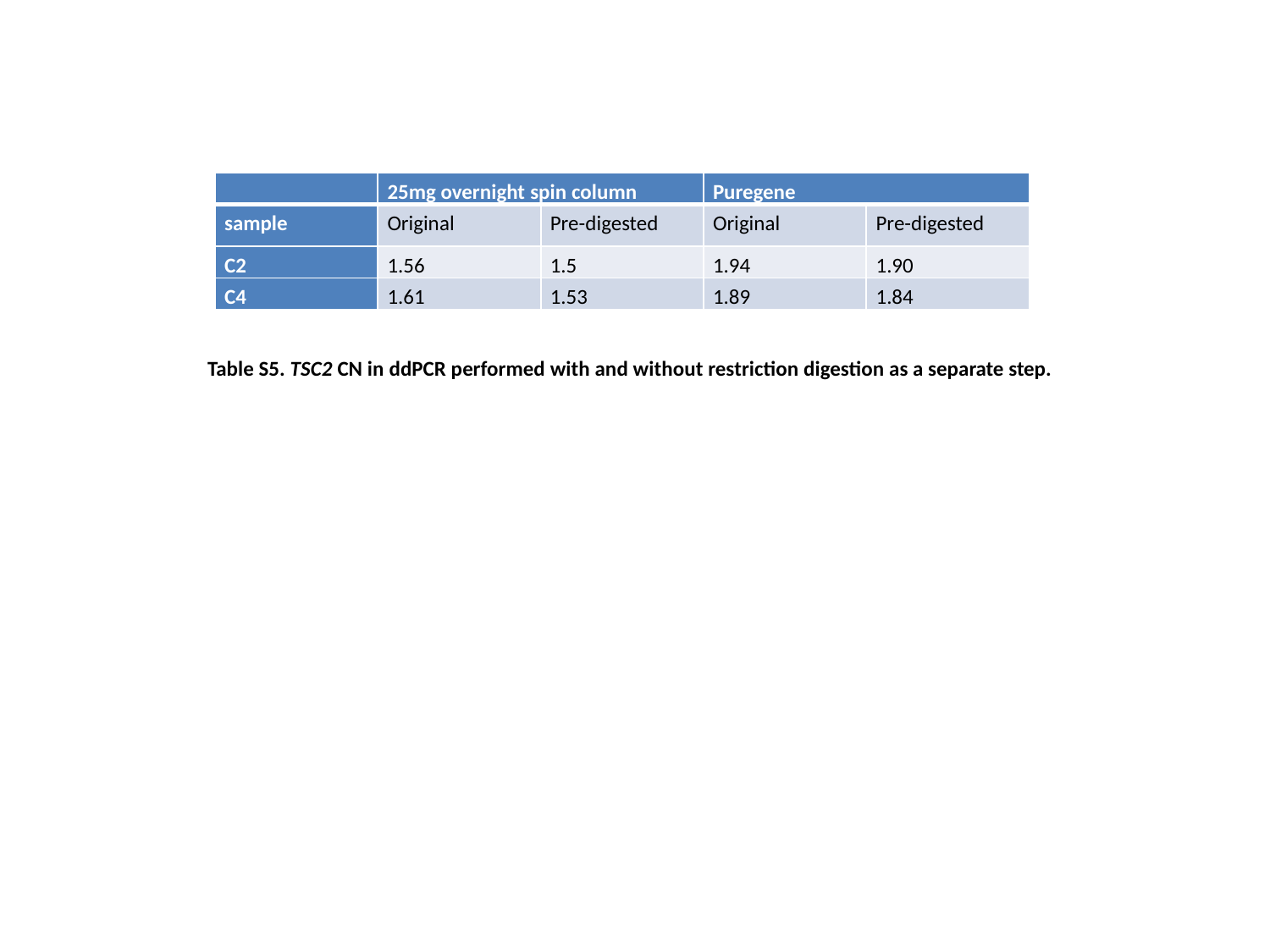

| | 25mg overnight spin column | | Puregene | |
| --- | --- | --- | --- | --- |
| sample | Original | Pre-digested | Original | Pre-digested |
| C2 | 1.56 | 1.5 | 1.94 | 1.90 |
| C4 | 1.61 | 1.53 | 1.89 | 1.84 |
Table S5. TSC2 CN in ddPCR performed with and without restriction digestion as a separate step.
