# Supplementary material for: DNA isolation protocol effects on nuclear DNA analysis by microarrays, droplet digital PCR, and whole genome sequencing, and on mitochondrial DNA copy number estimation
Source: PLoS One. 2017 Jul 6;12(7):e0180467. doi: 10.1371/journal.pone.0180467 (PMC5500342; doi:10.1371/journal.pone.0180467)
Supplement: S6 Table — (PPTX) [file pone.0180467.s018.pptx]

## Slide 1
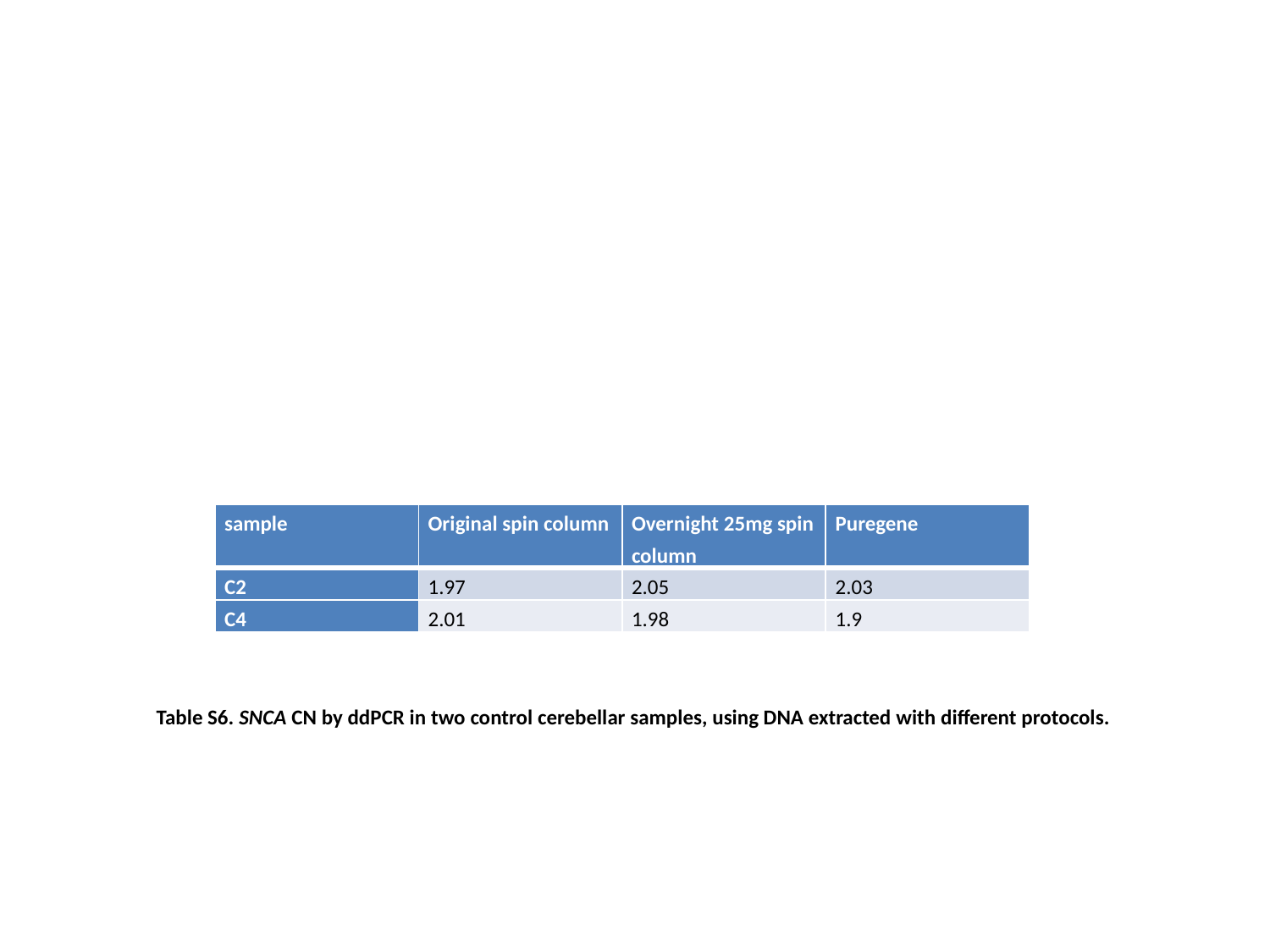

| sample | Original spin column | Overnight 25mg spin column | Puregene |
| --- | --- | --- | --- |
| C2 | 1.97 | 2.05 | 2.03 |
| C4 | 2.01 | 1.98 | 1.9 |
Table S6. SNCA CN by ddPCR in two control cerebellar samples, using DNA extracted with different protocols.
