# Supplementary material for: DNA isolation protocol effects on nuclear DNA analysis by microarrays, droplet digital PCR, and whole genome sequencing, and on mitochondrial DNA copy number estimation
Source: PLoS One. 2017 Jul 6;12(7):e0180467. doi: 10.1371/journal.pone.0180467 (PMC5500342; doi:10.1371/journal.pone.0180467)
Supplement: S7 Table — (PPTX) [file pone.0180467.s019.pptx]

## Slide 1
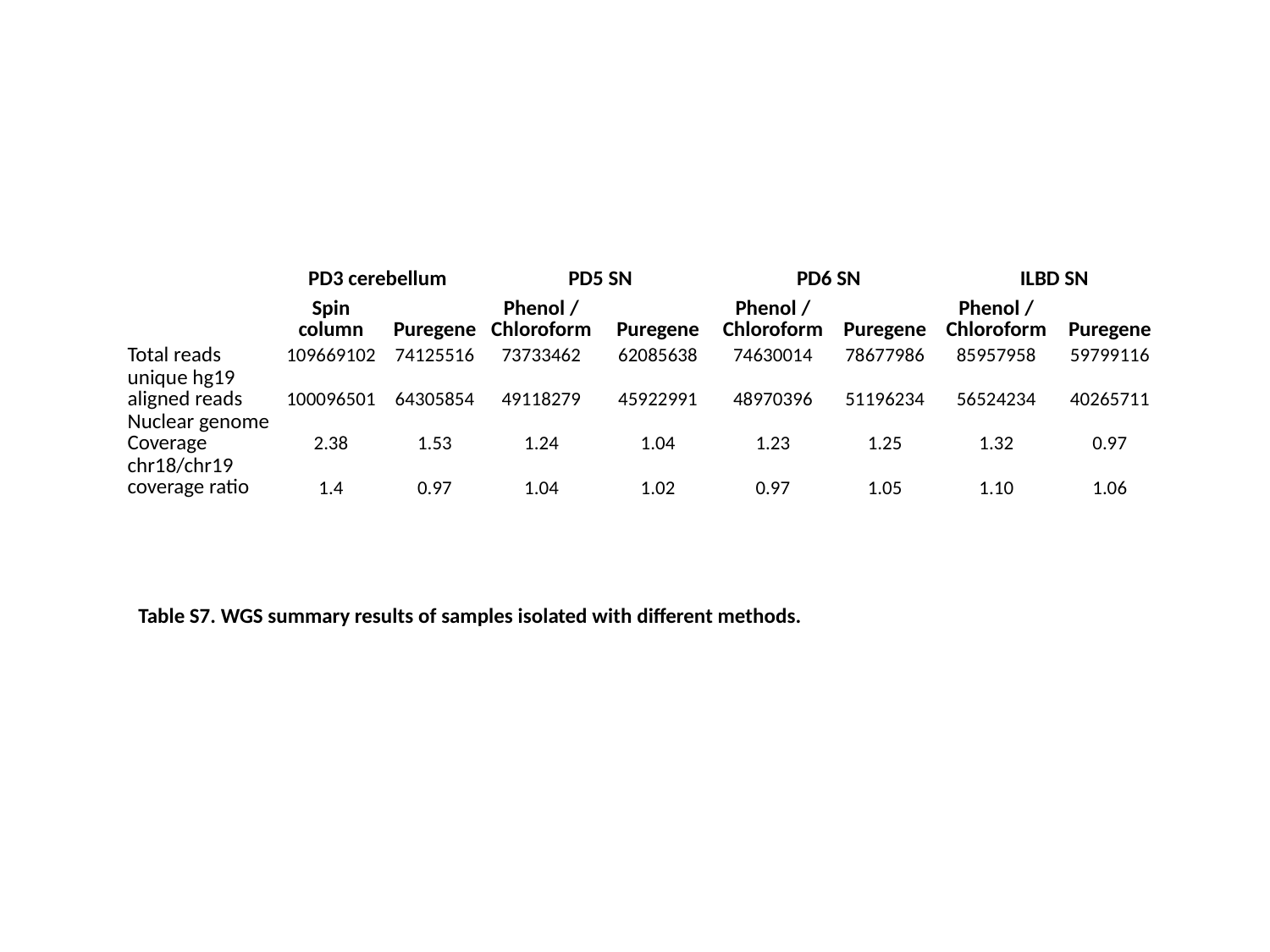

| | PD3 cerebellum | | PD5 SN | | PD6 SN | | ILBD SN | |
| --- | --- | --- | --- | --- | --- | --- | --- | --- |
| | Spin column | Puregene | Phenol / Chloroform | Puregene | Phenol / Chloroform | Puregene | Phenol / Chloroform | Puregene |
| Total reads | 109669102 | 74125516 | 73733462 | 62085638 | 74630014 | 78677986 | 85957958 | 59799116 |
| unique hg19 aligned reads | 100096501 | 64305854 | 49118279 | 45922991 | 48970396 | 51196234 | 56524234 | 40265711 |
| Nuclear genome Coverage | 2.38 | 1.53 | 1.24 | 1.04 | 1.23 | 1.25 | 1.32 | 0.97 |
| chr18/chr19 coverage ratio | 1.4 | 0.97 | 1.04 | 1.02 | 0.97 | 1.05 | 1.10 | 1.06 |
Table S7. WGS summary results of samples isolated with different methods.
