# Supplementary material for: DNA isolation protocol effects on nuclear DNA analysis by microarrays, droplet digital PCR, and whole genome sequencing, and on mitochondrial DNA copy number estimation
Source: PLoS One. 2017 Jul 6;12(7):e0180467. doi: 10.1371/journal.pone.0180467 (PMC5500342; doi:10.1371/journal.pone.0180467)

Supplementary note

As discussed in the main text, we felt that the extensive losses reported by both array types, which often overlapped, were very unlikely to be true mosaic losses if the SNP array B allele frequency (BAF) was not affected. As well as the genome-wide screen by HapLOH, we visually reviewed the BAF over several calls of losses. We noted that losses were often called in regions where few, or no, heterozygous SNPs were present (examples in S7 fig). We chose to attempt validation by PCR of the reported loss on chr1 involving *FBXO42* (S7a fig) where the aCGH probes also had mostly negative dLR over that region in the original spin column extraction. Furthermore, three other samples on the SNP array had a negative logR over this region, although a loss was not called (S7a fig).

The minimum loss size was 154.5 kb, too long to be amplified by PCR. Placing the primers just outside the borders of the predicted loss should allow us to amplify a product if the DNA indeed carries a loss. We designed two sets of primers using Primer-Blast (see table).

Primer pair	Orientation	Sequence	Theoretical wt product size (kb) and co-ordinates	Product size if deletion present	Annealing temperatures tried
1	F	GAATCTTCCCACACCCCTGG	156.2	6.4	65.2, 66.5, 67.8, **69.2**, 70.7
	R	CATGAGCCAGAAAGCAGCAC	16620804-16777048		
2	F	CTCCATCACACCAACCCCTC	156.4	6.6	66.5, 67.8, 69.2, **70.5**, 71.9
	R	CCAAGCATGAGCCAGAAAGC	16620625-16777053		

We used the high fidelity Phusion enzyme (NEB), with 100 ng genomic DNA in 25 µl total volume and 35 cycles in total, with an extension time of 2.5 minutes. We varied the annealing temperature, starting low, and increasing gradually, using human genomic reference DNA (Roche) and two other control cerebellar DNA samples not analysed before, to reach a point where no mis-priming products are seen. In both cases, we then used the annealing temperature at which faint non-specific bands were just visible for further experiments (in bold in the table), to ensure that the conditions were not too stringent for amplification of any correct products. We performed PCR at these conditions using 150 ng of cerebellar samples C1, C2, and C4, and did not see any correct products. For primer pair

2, we repeated the experiment with the GC buffer of the Phusion enzyme, and still saw no correct product (figure below).

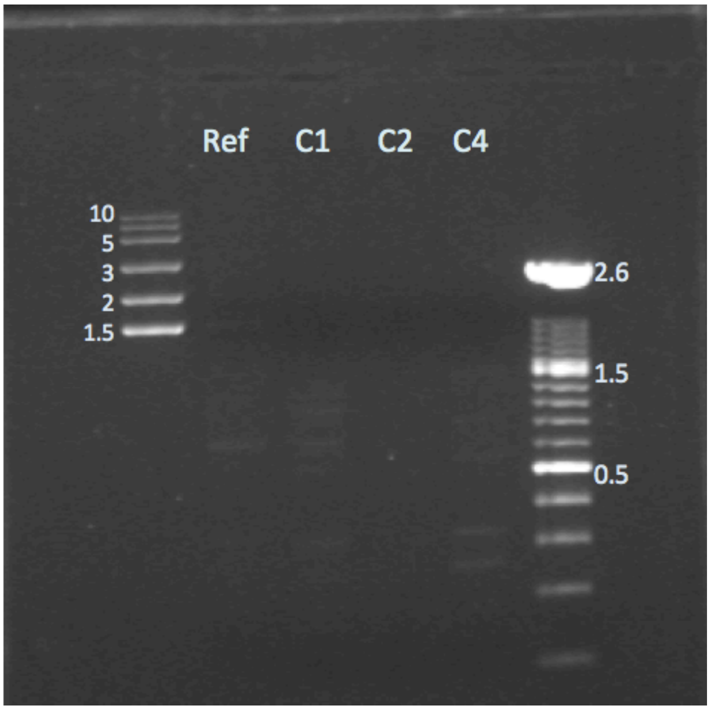

These results were consistent with our interpretation that the losses had been caused during DNA isolation, rather than deletions in the source tissue. We reviewed this region further in data from another sample (PD1) derived from aCGH experiments discussed in the main text, where we had hybridised cerebellar DNA against frontal cortex of the same brain, varying the extraction method. Probes over *FBXO42* had negative dLR when cerebellar DNA was hybridised against FC from the same brain, when both were extracted with spin columns (red line), as seen in the original C1 cerebellum v reference DNA (S7A fig). This was abolished when the same pair was hybridised after Puregene extraction (blue line). These data were also consistent with the subtle loss over this gene being extraction-induced.

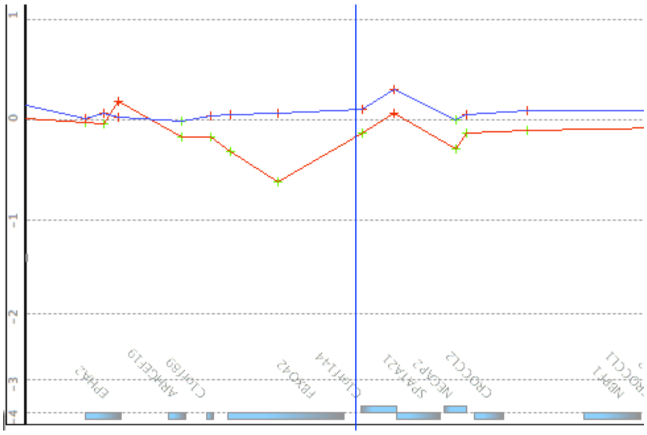

Supplement: S1 Note — (PDF) [file pone.0180467.s025.pdf]
