# Supplementary material for: DNA isolation protocol effects on nuclear DNA analysis by microarrays, droplet digital PCR, and whole genome sequencing, and on mitochondrial DNA copy number estimation
Source: PLoS One. 2017 Jul 6;12(7):e0180467. doi: 10.1371/journal.pone.0180467 (PMC5500342; doi:10.1371/journal.pone.0180467)
Supplement: S2 Fig — See also S5 Data. The chromosomal location, individual probe DLR with region of call highlighted, and CNV list in this region are shown for each. (PDF) [file pone.0180467.s002.pdf]

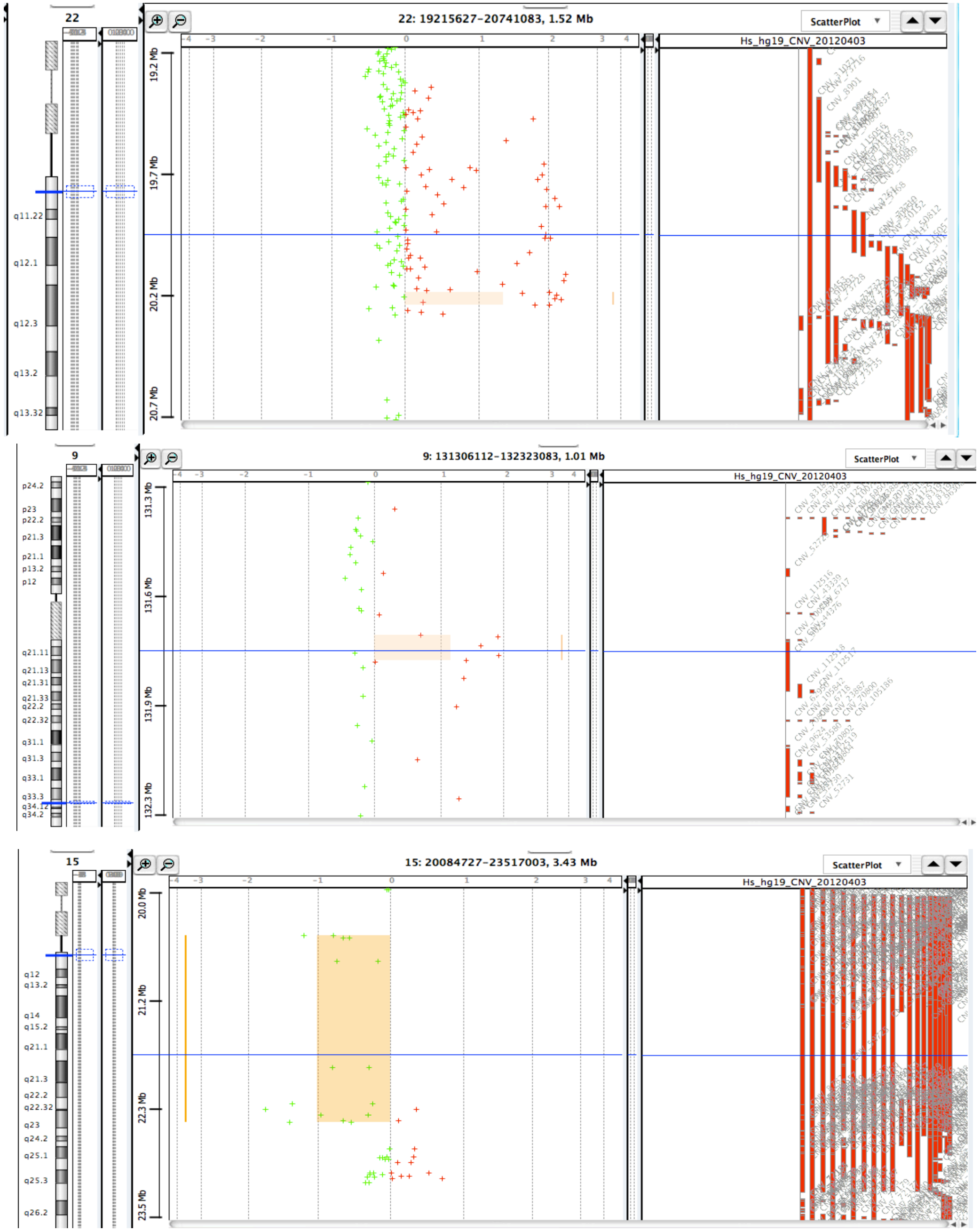
22
22: 19215627-20741083, 1.52 Mb
ScatterPlot
Hs_hg19_CNV_20120403
19.2 Mb
19.7 Mb
20.2 Mb
20.7 Mb
q11.22
q12.1
q12.3
q13.2
q13.32
9
9: 131306112-132323083, 1.01 Mb
ScatterPlot
Hs_hg19_CNV_20120403
131.3 Mb
131.6 Mb
131.9 Mb
132.3 Mb
p24.2
p23
p22.2
p21.3
p21.1
p13.2
p12
q21.11
q21.13
q21.31
q21.33
q22.2
q22.32
q31.1
q31.3
q33.1
q33.3
q34.2
15
15: 20084727-23517003, 3.43 Mb
ScatterPlot
Hs_hg19_CNV_20120403
20.0 Mb
21.2 Mb
22.3 Mb
23.5 Mb
q12
q13.2
q14
q15.2
q21.1
q21.3
q22.2
q22.32
q23
q24.2
q25.1
q25.3
q26.2
